# Multitasking Performance of Fe_3_O_4_/BaTiO_3_/Epoxy Resin Hybrid Nanocomposites

**DOI:** 10.3390/ma15051784

**Published:** 2022-02-26

**Authors:** Sevasti Gioti, Aikaterini Sanida, Georgios N. Mathioudakis, Anastasios C. Patsidis, Thanassis Speliotis, Georgios C. Psarras

**Affiliations:** 1 Smart Materials & Nanodielectrics Laboratory, Department of Materials Science, School of Natural Sciences, University of Patras, 26504 Patras, Greece; s.gioti@upnet.gr (S.G.); ksanida@upatras.gr (A.S.); patsidis@upatras.gr (A.C.P.); 2 Institute of Chemical Engineering Sciences (ICE-HT), Foundation for Research & Technology-Hellas (FORTH), Stadiou Str., Platani, P.O. Box 1414, 26504 Patras, Greece; mathioy@iceht.forth.gr; 3 Institute of Nanoscience and Nanotechnology, NCSR “Demokritos”, Aghia Paraskevi, 15310 Athens, Greece; t.speliotis@inn.demokritos.gr

**Keywords:** hybrid nanocomposites, thermomechanical behavior, electrical properties, energy storing/retrieving, magnetic response, multifunctionality

## Abstract

In this study, hybrid nanocomposites consisting of Fe_3_O_4_/BaTiO_3_/epoxy resin were prepared with varying amounts of filer content. Structural and morphological characterization, conducted via X-Ray Diffraction patterns and Scanning Electron Microscopy images, revealed the successful fabrication of composites and fine dispersion of inclusions. Thermomechanical properties are studied via Differential Scanning Calorimetry, Thermogravimetric Analysis, Dynamic Mechanical Analysis and static mechanical tests. Hybrid composites exhibit enhanced thermal stability and improved mechanical response. Indicatively, Young’s modulus, tensile strength and fracture toughness increase from 1.26 GPa, 22.25 MPa, and 3.03 kJ/m^3^ for the neat epoxy to 1.39 GPa, 45.73 MPa, and 41.08 kJ/m^3^ for the composites with 20 or 15 parts per hundred resin per mass (phr) of Fe_3_O_4_, respectively. Electrical behavior is investigated via Broadband Dielectric Spectroscopy and ac conductivity measurements. The real part of dielectric permittivity reaches the value of 11.11 at 30 °C for the composite with 40 phr of Fe_3_O_4_. The ability to store and retrieve electric energy on the nanocomposites is examined with the following parameters: the filler content and the applied voltage under dc conditions. Retrieved energy reaches 79.23% of the stored one, for the system with 15 phr of Fe_3_O_4_. Magnetic response is studied via a Vibrating Sample Magnetometer. Magnetic saturation, for the system with the highest magnetic filler content, obtains the value of 25.38 Am^2^/kg, while pure magnetic powder attains the value of 86.75 Am^2^/kg. Finally, the multifunctional performance of the nanocomposites is assessed regarding all the exerted stimuli and the optimum behavior is discussed.

## 1. Introduction

The impact of materials upon societies and everyday living has been high throughout the whole history of human beings and civilization. The continuously increasing demands of modern society for high-tech products and equipment impel the introduction of novel and advanced engineering materials. The vital role of materials in all technological applications, including but not limited to mechanical engineering, transportation, electrical and electronic engineering, energy, biomedical applications, and sports industry, appends requirements for light-weight, environmentally friendly, corrosion-resistant, thermo-mechanical-strengthened and low-cost engineering materials. Depending on the specific application optical clarity, electrical and magnetic behavior should be added. Moreover, all these properties must be present simultaneously in the same material and, according to the imposed stimuli, the suitable response or responses should be executed. By these means, the aspect of material/device is introduced and multifunctionality appears as an emerging request [1,2,3].

Functional materials represent a unique class of engineering materials that have the ability to perform certain functions (operations) when they are exposed to an external stimulus or control signal. Interestingly, functional materials retain their performance even if their volume is subdivided [4]. Materials able to reversibly convert energy from one kind to another are considered active functional materials. The occurring research challenge is the development of a composite material/device being able to execute several functions (such as variable polarization, tunable dielectric response, adjustable conductivity, varying magnetic performance, energy and memory storage, etc.) while being easy to make, light-weight, and cost-effective and exhibiting at the same time structural integrity and a suitable thermal response. By selecting suitable constituents and a suitable fabrication procedure, novel hybrid composites could be prepared, in which multifunctionality should be integrated and multitasking performance should characterize the developed material/device.

**Figure 1:**
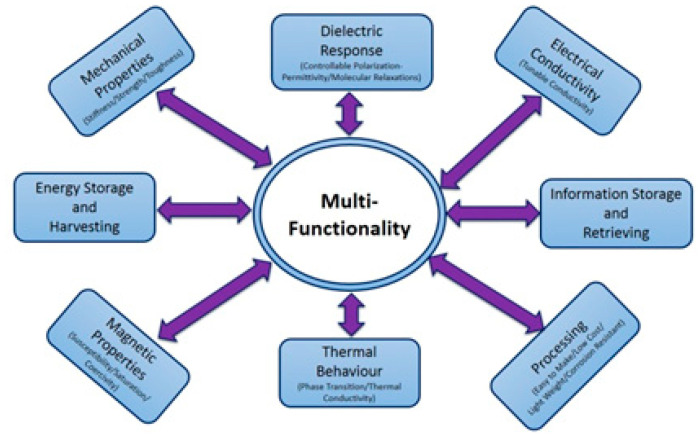
Schematic representation of materials’ multifunctionality.

Until recently, the choice of engineering materials for a specific application was based only on their mechanical and physical properties, such as Young’s modulus, strength, conductivity, etc., which constitute their behavior at service. Nowadays, engineering materials should be able to respond in real time to a rapidly varying environment. Thus, besides the nominal values of various properties of materials, their functionality and their controllable behavior under different conditions and stimuli are of great importance and constitute the main goal of novel materials. However, monolithic materials do not exhibit the required versatility in their performance, and new composite materials or materials’ systems should be designed and developed. Multitasking performance results by combining different desirable properties/responses in a material’s system. Various loading conditions, at service, activate the corresponding behavior each time. Mechanical sustainability, suitable thermal response, tunable electric conductivity, variable electric polarization and dielectric permittivity, magnetic properties, and thermally induced phase changes could be parts of the overall multifunctional behavior [4,5,6]. Future emerging applications for multifunctional nanocomposites could include wearable and implantable devices, autonomous robotics, and prosthetics and health care systems. Figure 1 presents a schematic representation of multifunctional behavior.

Polymer matrix composites and nanocomposites are considered an important class of engineering materials mostly because of their thermo-mechanical properties [7,8,9,10]. The improved mechanical behavior of nanocomposites in tandem with their low weight are key features for applications in the fields of aerospace, the automobile industry and in structural constructions. Moreover, their shape can be fitted according to the needs of the application, and their optical, electrical, and magnetic properties can be tailored by controlling the type and the amount of the reinforcing phase, as well as the manufacturing method. Although most of the research work conducted in the field of nanocomposites is focused on mechanical properties, recently a lot of attention has been given to their electrical, magnetic, and functional behavior [11,12,13,14,15]. Thus, polymer matrix composites form an adequate type of material for integrating multifunctionality.

In this study, a typical thermosetting epoxy resin is employed as the matrix, because of its high corrosion resistance, low humidity absorption, thermo-mechanical response and stability, and its ease of processing and commercial availability at a low cost. Magnetite (Fe_3_O_4_) nanoparticles are used as the first reinforcing phase. Magnetite is one of the most common iron oxides, exhibiting the strongest magnetic behavior between all the transition metal oxides [16,17]. Fe_3_O_4_ has an inverse spinel structure according to the Fd-3m space group, with the Fe(III) ions to be randomly sited between octahedral and tetrahedral lattice points and Fe(II) ions in octahedral points [18,19,20]. Iron oxides, because of their magnetic properties, are used in many wide-ranging technological applications. Recording media, catalysts, ferrofluids, magnetic inks, targeting drug delivery and biomedical devices are some of them [16,17,18,19,21,22,23,24]. The second reinforcing phase employed for the fabrication of the studied hybrid nanocomposites is barium titanate micro-particles (BaTiO_3_). Barium titanate is a polymorphic material undergoing transitions from the R3m rhombohedral to Amm2 orthorhombic to P4mm tetragonal and to Pm3m cubic crystal structure at approximately −90, 5, and 130 °C, respectively [22,23,24,25,26,27,28]. Moreover, as a typical ferroelectric material, barium titanate undergoes a structural transition from the tetragonal polar ferroelectric phase to the cubic non-polar paraelectric phase, at a critical temperature (*T_C_*), known as the Curie temperature [6,23,24,25,26,27,28,29]. Critical temperature in ceramic BaTiO_3_ lies in the range of 120 to 130 °C and appears to be dependent on the particles’ size [6,22,23,24,25,26,27,28]. Ferroelectric to paraelectric phase change is a first-order transition characterized by a discontinuous change of polarization at the critical temperature [29]. BaTiO_3_, due to its high dielectric permittivity, ferroelectric properties and non-toxicity, is used in electronic and microwave devices, as an interlayer in ceramic capacitive structures, as a sensor, in portable energy storage systems, in supercapacitors, etc. Recent studies in BaTiO_3_/polymer nanocomposites have opened new opportunities for future applications in the fields of portable energy devices, triboelectric micro/nano-generators, flexible micro/nano-electro-mechanical systems (MEMS/NEMS), implantable devices, and as additives for bone generation [30,31,32,33]. Several studies [23,24,25,26,27,28,29] have shown that the ferroelectricity of barium titanate particles reduces with their size. Below *T_C_*, at the nanoscale level, the tetragonality of BaTiO_3_ decreases or even completely vanishes, since the aspect ratio c/a of the unit cell dimensions approaches unity. It has been found that, below critical temperature, tetragonal and cubic structures co-exist in barium titanate nanoparticles, leading to a weakness of the ferroelectric effect and to a considerable reduction in unit cell’s polarizability [23,24,25,26,27,28,29]. Thus, the ferroelectric-to-paraelectric transition is difficult to observe in BaTiO_3_ nanoparticles [27,28,29]. For this reason, in the present study, microparticles of barium titanate have been employed. The constant content of BaTiO_3_ particles results from previous studies of our group and corresponds to the optimum concentration in BaTiO_3_/epoxy composites related to their dielectric response and functional behavior [6,26,34]. In the fabricated hybrid composites, the content of magnetite nanoparticles varies, aiming to study the influence of their concentration upon the induced magnetic properties and the synergy with barium titanate in dielectric response, ac conductivity, electrical energy storage and retrieving, static and dynamic mechanical behavior, and thermal properties. Prior to the aforementioned experimental investigation, structural and morphological characterization of the fabricated systems was conducted by means of X-Ray Diffraction patterns (XRD) and Scanning Electron Microscopy images (SEM), respectively.

## 2. Materials and Methods

In the present study, a set of epoxy-based hybrid nanocomposites were prepared and studied. A low-viscosity, two-component epoxy resin system was used as polymer matrix. Specifically, epoxy prepolymer and curing agent with the trade names Epoxol 2004 A and Epoxol 2004 B, respectively, were purchased by Neotex SA (Athens, Greece). Barium titanate (ΒaTiO_3_) particles with diameter less than 2 μm and iron oxide (magnetite, Fe_3_O_4_) nanoparticles with diameter in the range of 50–100 nm, were used as reinforcing phases. Both fillers were supplied by Sigma-Aldrich.

The fabrication process started by mixing pre-calculated amounts of barium titanate and iron oxide particles into resin prepolymer, at ambient temperature. Afterwards, the mixture was stirred using a sonicator (Elma S30H, Elmasonic, operating at sweep mode at 280 W) for 10 min at *T* = 50 °C, in order to achieve a smooth dispersion of particles into the liquid resin and avoid the formation of agglomerations that leads to degradation of the properties of the composite. Once the stirring process was completed, the mixture was left at room temperature for a while for reducing its temperature. Then, the curing agent was added in the mixture at a 2:1 *w*/*w* mixing ratio of the epoxy prepolymer and the curing agent. The resulted mixture was sonicated, at ambient temperature, for 10 min. Eventually, the mixture was poured into silicone molds and cured at room temperature for 7 days. Finally, prepared specimens were post-cured for 4 h at *T* = 120 °C. Sets of specimens were fabricated at suitable geometries for each employed experimental technique. The barium titanate content was kept constant at 10 phr (parts per hundred resin per mass) in all fabricated nanocomposites, while the concentration of magnetite was a varying parameter taking the values: 5, 10, 15, 20, 40, and 50 phr. The amount of the BaTiO_3_ content was chosen by evaluating previous studies from our group [6,26,34]. For comparison reasons, an unfilled epoxy specimen was also prepared. The content of all fabricated systems is listed in Table 1.

Structural characterization of the prepared hybrid composites was conducted with X-Ray Diffraction (XRD) patterns. XRD patterns were obtained using a Bruker AXS D8 Advance (Coventry, UK) device with Bragg–Brentano geometry. The used detector and the incident radiation spectral line were LynxEye and Cu Kα (*λ* = 1.54062 Å), respectively. Scan mode was continuous, with a 0.02° 2θ step and 0.5 s/step scan speed. Source slit was 0.6 mm, while voltage and current were at 40 kV and 40 mA, respectively.

The quality of the dispersion of particles in the epoxy matrix, as well as the morphology of the produced specimens, was examined by means of Scanning Electron Microscopy (SEM) via a Carl Zeiss EVO MA 10 apparatus.

Differential Scanning Calorimetry (DSC) was employed in order to investigate the thermal response of the nanocomposites via a TA Q200 device provided by TA Instruments. A few mg from each nanocomposite were put into a shielded aluminum crucible and an empty crucible was used as reference. Specimens were tested in the range from 20 to 100 °C with a temperature ramp of 5 °C/min. Thermogravimetric Analysis (TGA) was used for the investigation of thermal degradation of the examined systems by employing a TA Q500 device (TA Instruments). Measurements were conducted from ambient temperature up to 600 °C with 10 °C/min heating rate. Dynamic mechanical behavior was assessed by means of a Dynamic Mechanical Analysis (DMA) with a TA Q800 apparatus supplied also from TA Instruments. The type of the used test was three-point bending, the applied temperature range was from ambient temperature to 100 °C, at a 5 °C/min rate, and the frequency of the exerted oscillating mechanical stimulus was 1 Hz.

The static mechanical properties of the prepared nanocomposites were investigated by means of an Instron 5582 tester, at ambient temperature and at 5 mm/min tension rate.

Electrical properties, i.e., dielectric response and ac conductivity were studied by means of Broadband Dielectric Spectroscopy (BDS). Experimental setup is composed by an Alpha-N Frequency Response Analyzer, Phecos System, BDS 1200 dielectric cell, and windeta software. All components of the dielectric setup were provided by Novocontrol Technologies. Measurements were conducted under isothermal conditions in the temperature range 30–160 °C. The frequency of the applied field varied between 10^−1^ and 10^7^ Hz, with *Vrms* = 1 V. The increasing temperature step, after each frequency scan, was 5 °C. Ac dielectric measurements were conducted according to the ASTM D150 specifications.

The ability of storing and retrieving energy was investigated via dc measurements, by recording the time-dependent charging and discharging currents. For this reason, a High-Resistance Meter dc (Agilent 4339B) device was employed. Experimental tests include recording, consecutively and in real time, the charging and discharging currents as a function of time. Measurements were conducted at ambient temperature with three different applied voltage levels, namely, 50, 100, and 150 V. Tested samples were put between the electrodes of a parallel-plate capacitor. In all cases, the charging time was 60 s. To avoid the presence of any pre-stored charges in the specimens, a discharging short-circuit procedure was employed prior of every measuring sequence. Dc tests were made according to the ASTM D257 specifications. Analytical description of the experimental setup and procedure can be found in [29,35].

Finally, the magnetic properties of the hybrid nanocomposites were examined by employing a Vibrating Sample Magnetometer (VSM, Princeton Applied Research) at ambient. Applied magnetic fields were ranging from −20 to 20 kOe.

## 3. Results

**Figure 2:**
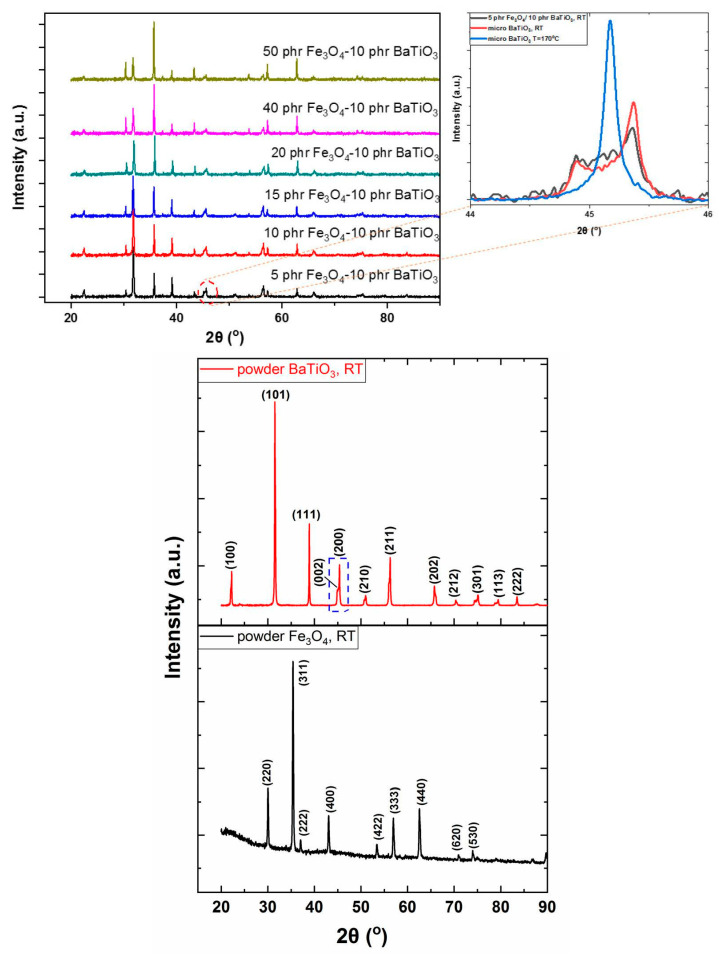
XRD patterns of all studied hybrid composites and the employed fillers at room temperature (RT). Comparative XRD patterns from barium titanate micro-particles at RT and 170°C and from the 5 phr Fe_3_O_4_/10 phr BaTiO_3_/epoxy composite at RT.

Figure 2 presents XRD patterns of all the fabricated systems, as well as of the two employed reinforcing phases. Recorded diffraction peaks in composites are consisted with the magnetite and barium titanate powder diffraction patterns, while peak intensity increases steeply with filler content. Both attributes denote the successful incorporation of ceramic particles in the epoxy matrix.

The inverse cubic spinel crystal structure of Fe_3_O_4_ particles corresponds to the Fd-3 m space group with a lattice parameter a = 8.3582 Å. Characteristic diffraction peaks of Fe_3_O_4_, related to the planes (220), (311), (400), (422) and (440) are detected in the recorded patterns [13,14,36,37]. BaTiO_3_ diffraction patterns include the characteristic peaks of (100), (101), (111), (002), (200), (210) and (211). The employed microparticles of barium titanate are, at ambient temperature, tetragonal in their crystal structure. The latter becomes evident by the formation of two distinct peaks in the 2θ range 44–46°, which correspond to the (002) and (200) diffraction planes [6,29,36,38,39,40,41]. These characteristic peaks are present in the patterns of all nanocomposites revealing the ferroelectric phase of the BaTiO_3_ particles, at room temperature. At temperatures higher than *T_C_*, micro-BaTiO_3_ particles revert from the tetragonal lattice and ferroelectric phase to the cubic lattice and paraelectric phase and then only the (200) peak is present in the same 2θ range [6,24,29,40,41]. The XRD patterns on the right graph of Figure 2 present patterns of BaTiO_3_ particles at room temperature and 170 °C, far below and far above *T_C_*, respectively, compared with the pattern of the 5 phr Fe_3_O_4_/10 phr BaTiO_3_ reinforced composite. Interestingly, the two peaks of the tetragonal structure are formed in both the powder’s and the composite’s patterns at room temperature. At 170 °C, the cubic phase becomes evident by the formation of a single peak. Figure S1a, in Supplementary Information, presents an energy-dispersive X-ray spectroscopy spectrum for the composite with 5 phr Fe_3_O_4_/10 phr BaTiO_3_ content, where the presence of Fe, Ba and Ti is evident.

**Figure 3:**
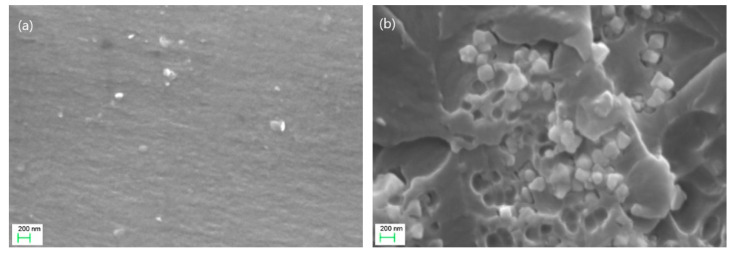
SEM images from the composites: 5 phr Fe_3_O_4_/10 phr BaTiO_3_ (**a**) and 40 phr Fe_3_O_4_/10 phr BaTiO_3_ (**b**). The indicated scale bar corresponds to 200 nm.

The morphology of the studied systems was assessed via SEM images. Representative images of cryo-fractured surfaces from the 5 phr Fe_3_O_4_/10 phr BaTiO_3_ and 40 phr Fe_3_O_4_/10 phr BaTiO_3_ systems are shown in Figure 3a,b, respectively. In all cases, fine dispersions of the magnetite nanoparticles and the microparticles of barium titanate are present. The successful fabrication of the hybrid composites is verified by the absence of voids and agglomerates. A limited number of small clusters can be detected at the high fillers’ concentration systems. Figure S1b, in Supplementary Information, shows an SEM image from the composite with the highest filler content (50 phr Fe_3_O_4_/10 phr BaTiO_3_), at a lower magnification, where fine dispersions and small clusters can be observed.

DSC thermographs were used for the determination of glass transition temperature (*T_g_*) for each examined system. *T_g_* was evaluated via the point of inflection of the endothermic step-like increase in specific heat capacity in the recoded spectra, via suitable software supplied by TA Instruments. The obtained values of *T_g_* are shown in Table 1. Obtained values do not vary significantly with filler content, indicating balanced interactions between the ceramic particles and between particles and matrix. It is well established in the literature that the increase in glass transition temperature with filler content corresponds to strong interactions at the boundaries of the particles with the macromolecules and also to weak interactions between the inclusions. In contrast, diminishing the values of *T_g_* with filler content indicates strong interactions between the particles and weak interactions at the polymer/filler interface [9,42].

**Figure 4:**
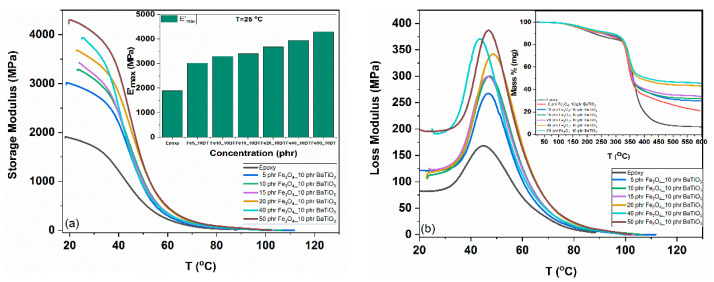
Storage modulus (**a**) and loss modulus (**b**) as a function of temperature for all studied systems. Left inset max E’ versus filler content. Right inset TGA thermographs.

Thermomechanical behavior for all the studied systems, assessed via DMA data, is depicted in Figure 4. Figure 4a presents the variation of storage modulus versus temperature varying the reinforcing phase content. All curves exhibit a variation from high to low values with the increase in temperature, denoting the transition from the rigid glassy state to the viscous rubbery-like one. The strengthening ability of the Fe_3_O_4_ nanoparticles is apparent in the glassy state from the systematic increase in storage modulus values with magnetite content. This behavior is further illustrated in the bar chart, inset of Figure 4a, which depicts the maximum values of storage modulus as a function of magnetite content and is considered a strong indication of fine particles’ dispersion and good adhesion between the constituents [43,44]. Loss modulus spectra as a function of temperature for the same set of systems are presented in Figure 4b. In the previously mentioned transition zone, loss peaks are formed corresponding to the dissipation as heat of mechanical energy. Peaks are used for the determination of the onset of the glass transition temperature range [45]. Determined values are shown in Table 1.

The thermal stability of the examined systems was studied by recording the TGA degradation thermographs shown in the inset of Figure 4b. Recorded spectra include two mass loss mechanisms. The first one, observed in the range of 150 to 250 °C, is related with the breakdown of unreacted epoxy rings and possibly to the existence of impurities. The second one in the range of 300 to 400 °C reflects the decomposition of the matrix. Ceramic nanoparticles are beneficial to the thermal stability of the nanocomposites, since the range of occurrence of the first process shifts to higher temperatures. In Table 1, the temperature corresponding to the 5% initial mass loss, for all studied systems, is listed.

**Figure 5:**
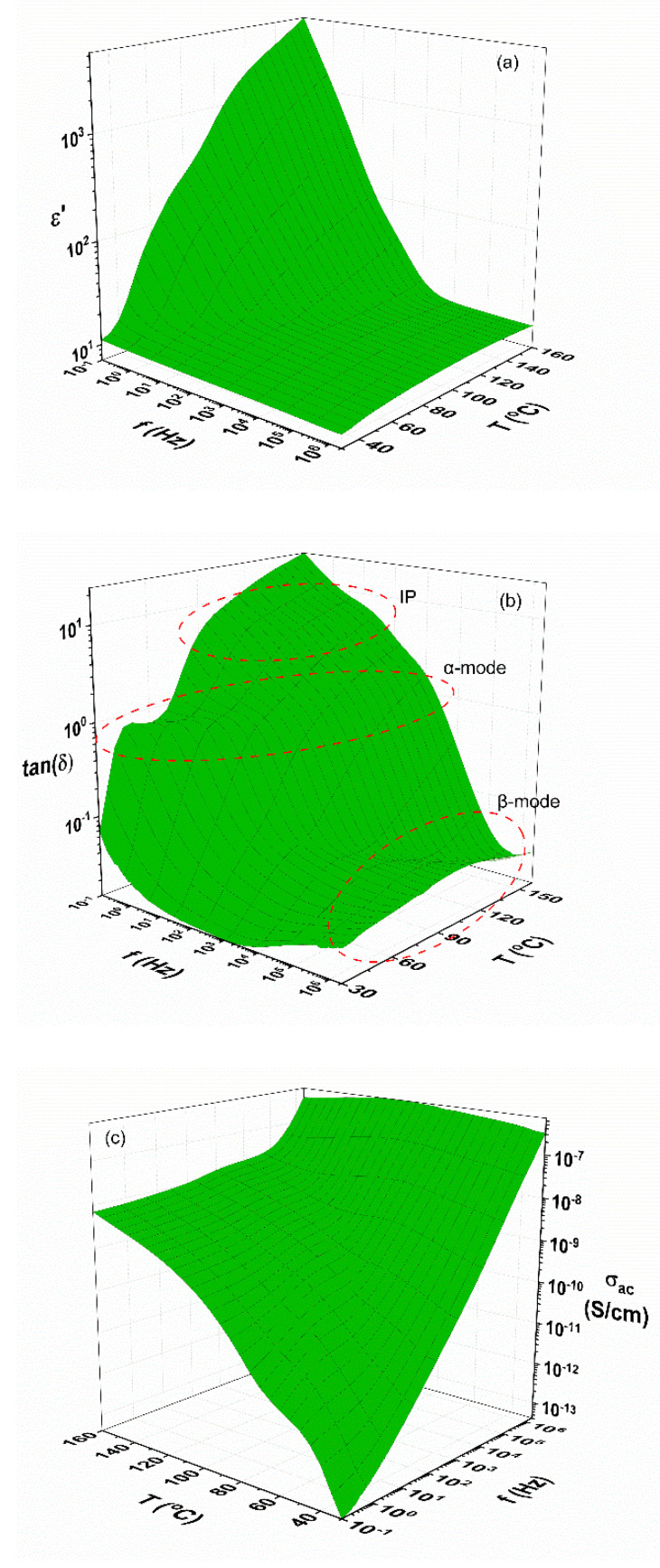
(**a**) Real part of dielectric permittivity, (**b**) loss tan*δ*, and (**c**) *σ_ac_* as a function of frequency and temperature for the 40 phr Fe_3_O_4_/10 phr BaTiO_3_/epoxy hybrid composite.

The dielectric response of the examined systems is depicted in the representative three-dimensional (3D) graphs of the 40 phr Fe_3_O_4_/10 phr BaTiO_3_/epoxy composite in Figure 5. All systems exhibit similar behavior. Figure S2, in Supplementary Information, presents another example for the 10 phr Fe_3_O_4_/10 phr BaTiO_3_/epoxy composite. Figure 5a presents the variation of the real part of dielectric permittivity (*ε*′) with temperature and frequency. The influence of temperature is more pronounced in the low-frequency range, resulting in higher values of *ε*′. In this range, the alternation of the applied electric field is slow, thus providing the needed time to the dipoles (permanent and induced) to follow the field. Temperature acts as an additional facilitating parameter of the dipoles’ orientation by thermal agitation. In the same range (high-temperature and low-frequency era), space charges are built up at the composite’s interface, forming induced dipoles with dimensions similar to those of the employed particles size. The accumulated charges at the constituents’ interface contribute to Interfacial Polarization (IP) and thus to the real part of dielectric permittivity and potentially to conductivity by migrating through the interface. In the middle frequency and temperature region, spectra of *ε*′ exhibit a transition from higher to lower values, indicative of the presence of a relaxation process. This transition shifts to higher frequencies as temperature rises. At the high-frequency edge of the recorded spectra, the real part of dielectric permittivity acquires its lower values since dipoles’ inertia obstructs their orientation parallel to the field. Figure S3, in Supplementary Information, depicts *ε*′ versus temperature for all studied systems at 0.1 Hz. Hybrid composites attain significantly higher values, above 120 °C, compared to the neat epoxy, denoting the contribution of interfacial polarization to the real part of dielectric permittivity.

Spectra of the loss tangent (tanδ) versus frequency and temperature are shown in the 3D diagram of Figure 5b. In the recorded spectra, three relaxation mechanisms can be identified. In the low-frequency, high-temperature zone, the observed mechanism corresponds to IP and is the slowest process (higher relaxation time) of all three. In the mid-zone, the occurring process reflects the glass-to-rubber transition of the epoxy matrix (α-relaxation), and in the high-frequency zone the occurring weak mechanism is attributed to the re-alignment of side polar-groups of the main polymer chain (β-relaxation). This process is the faster one (lower relaxation time). The three-dimensional graph of Figure 5c presents ac conductivity as a function of frequency and temperature for the same nanocomposite. Values of ac conductivity have been calculated according to Equation (1), and *σ_ac_* represents the sum of all dissipative processes comprising the effect of space charge migration and dipoles’ orientation [46].

(1) $$\sigma_{ac}\left(\omega \right)=\varepsilon_{0}\omega \varepsilon^{\prime \prime }$$

where $\varepsilon_{0}$ is the permittivity of free space, ω the angular frequency of the applied field and *ε*″ the imaginary part of dielectric permittivity or the dielectric loss index.

The obtained profile of ac conductivity is characteristic of a non-conductive material. Specifically, ac conductivity is highly influenced by temperature in the low-frequency zone, since the thermally activated charge carriers acquire sufficient time and energy to migrate because of the slow alternation of the applied field. In this regime, the participating number of carriers is limited, although charges cover relative longer distances within the nanocomposite. The insulating matrix exerts potential barriers to carriers and conductivity attains rather constant values, limiting its dc value. On the contrary, above a critical frequency, *σ_ac_* augments exponentially with frequency. In this region, temperature agitation appears not be effective and an increased number of charges migrate forward and back between contiguous sites, because of the high field’s frequency.

## 4. Discussion

**Figure 6:**
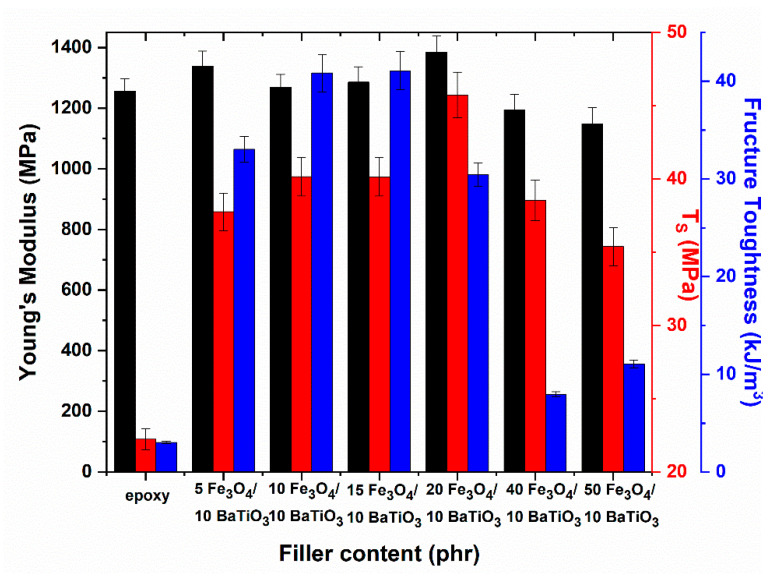
Young’s modulus, tensile strength (*T_s_*), and fracture toughness for all studied systems.

Results from the static mechanical tests are shown in Figure 6. Young’s modulus, tensile strength and fracture toughness were all elaborated from the tensile stress–strain plots. Modulus of elasticity initially increases with iron oxide content, up to 20 phr, and then declines with further content increase. The effect of filler content on tensile strength and fracture toughness is remarkable, especially up to the 20 phr Fe_3_O_4_/10 phr BaTiO_3_ nanocomposite. At even higher filler content, values of tensile strength and fracture toughness diminish significantly. It is possible, that the formed small clusters act as stress concentration points. In general, the mechanical endurance of nanocomposites increases with magnetite and, although not directly comparable, it seems to be in accordance with the data from the DMA tests.

**Figure 7:**
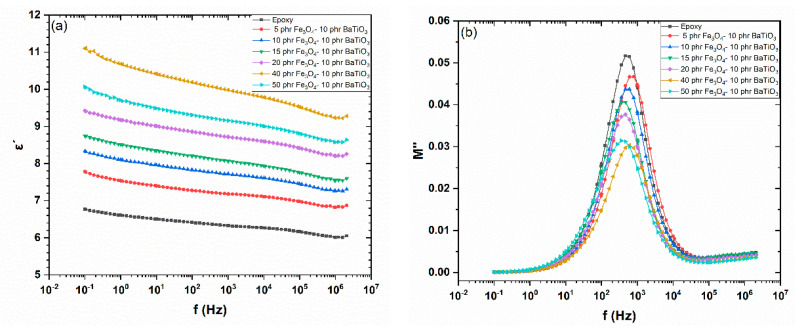
(**a**) Real part of dielectric permittivity as a function of frequency at 30 °C, and (**b**) loss modulus index as a function of frequency at 160 °C, for all studied systems.

The real part of dielectric permittivity versus frequency, at 30 °C, for all studied systems is presented in Figure 7a. The variation of *ε*′ with filler content reveals an additional aspect of the reinforcing ability of magnetite nanoparticles. Its values increase systematically with filler content up to the specimen with 40 phr Fe_3_O_4_/10 phr BaTiO_3_. The composite with the highest filler content exhibits lower values than the previously mentioned one. At this high filler content (50 phr Fe_3_O_4_/10 phr BaTiO_3_), the formation of clusters and agglomerates is possible, leading to a reduction in interfacial area between the reinforcing phase and matrix, which in most cases causes a reduction in the real part of permittivity. In addition, the increased concentration of particles exerts spatial obstructions to the orientation of dipoles with the same result. The electric modulus loss index (*M*″) as a function of frequency, at 120 °C, is shown in Figure 7b. Recorded peaks correspond to the glass-to-rubber transition of the polymer matrix. Peaks’ location is indicative of the glass transition temperature, and the shifting of the peaks to higher frequencies reflects a decrease in *T_g_*, while peaks’ shift to lower frequencies denotes an increase in *T_g_* [9,42]. Figure 7b suggests qualitatively that glass transition temperature does not vary significantly with filler content, being in accordance with the results from the thermal analysis.

Dielectric Reinforcing Function (DRF) provides an additional method for studying the dielectric response of composite materials. DRF is defined via Equation (2) as the ratio of composite’s *ε*′ upon the respective one of the matrix [47]. DRF neglects the influence of geometrical characteristics, expresses the ability of the employed filler in strengthening the composite’s dielectric properties, is a measure of the normalized polarization, and reflects the potential for storing electrical energy.

**Figure 8:**
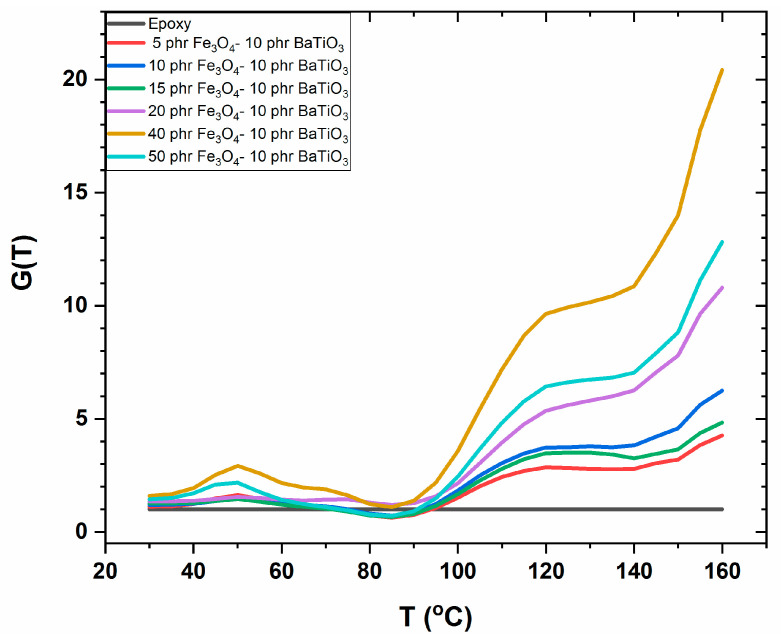
DRF, at 0.1 Hz, versus temperature for all the examined hybrid composites.

Dielectric reinforcing function is defined as [6,42]:

(2) $$G\left(f,T\right)=\frac{{\varepsilon^{\prime }}_{com}\left(f,T\right)}{{\varepsilon^{\prime }}_{mat}\left(f,T\right)}$$

where ${\varepsilon^{\prime }}_{com}\left(f,T\right)$ and ${\varepsilon^{\prime }}_{mat}\left(f,T\right)$ correspond to the real part of dielectric permittivity for the composite and the pure matrix at frequency *f* and temperature *T*. The temperature dependence of DRF at 0.1 Hz for all the examined hybrid composites is depicted in Figure 8. In the vicinity of 120 °C, a hump is observed in all DRF spectra, being indicative of the critical temperature (*T_C_*) of the ferroelectric-to-paraelectric transition of BaTiO_3_ particles (occurring in the range of 120 to 130 °C) [6,29]. In the same temperature zone, IP is present, providing high values of the real part of dielectric permittivity. The coexistence of both effects results in broad step-like peaks and in values of *G*(*f*,*T*) that further increase with temperature, because of the enhanced mobility of dipoles.

**Figure 9:**
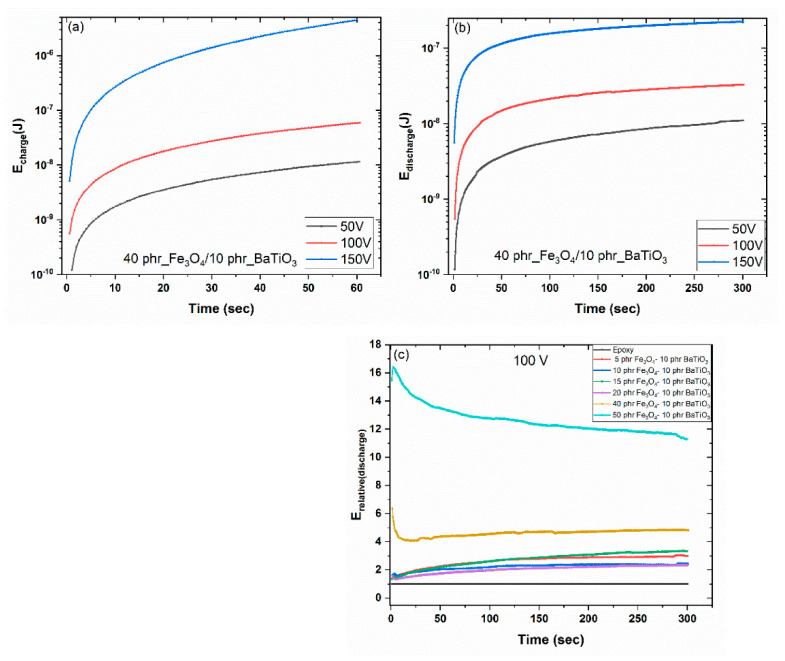
Charging (storing) energy (**a**), discharging (retrieving) energy (**b**), and relative discharging (retrieving) energy (**c**), as a function of time at room temperature.

The amount of the stored and retrieved energy and the efficiency of this procedure was investigated by integrating the time-dependent charging/discharging current functions, via Equation (3):

(3) $$E=\frac{1}{2}\frac{Q^{2}}{C}=\frac{1}{2}\frac{{\left[\int I\left(t\right)dt\right]}^{2}}{C}$$

where *E* is the stored/retrieved energy at the composite, *Q* is the amount of charge, *I*(*t*) is the charging or discharging current and *C* is the capacitance of the composite as derived by the BDS measurements [35,48,49]. The stored and retrieved energies for the composite with 40 phr Fe_3_O_4_/10 phr BaTiO_3_ reinforcing phase content, for all three examined voltage levels, are presented in Figure 9.

Both energies increase with the level of charging voltage. The applied voltage/electric field injects charge carriers that cannot percolate the whole specimen because of its insulating nature. The epoxy matrix exerts potential barriers and charge carriers should attain increased activation energy to overcome these barriers. Since charge migration in dielectric systems is mostly a thermally activated process, at ambient temperature a limited number of charges is able to overcome the local barriers. The majority of the free charges are trapped at the interface between the constituents of the composite, resulting in limited conduction and enhancement of IP. Polymer nanocomposites can act as a network of nanocapacitors, since nanoinclusions, which are distributed within the insulating matrix, can be exploited as energy-storing elements. Energy can be stored and harvested in this network in a fast charge/fast discharge procedure defining an active device on the nanoscale. However, the application of higher voltage/electric field during the charging process lowers the potential barriers, increasing the mobility of charges, which follow a trapping/detrapping sequence while migrating through the extensive interfacial area of the nanocomposite. This procedure leads to an increment of conductivity and leakage currents, diminishing in some cases the retrieved energy [48,49]. The effectiveness of the storing/retrieving process is appraised by introducing the coefficient of energy efficiency (*n_eff_*), which is defined via Equation (4):

(4) $$n_{eff}=\frac{E_{retrieved}}{E_{stored}}$$

where $E_{retrieved}$ and $E_{stored}$ are the retrieved and stored energies, respectively. Important parameters for *n_eff_* are the charging voltage and charging/discharging instant of time [35,48].

Table 2 lists the values of *n_eff_* for all three exerted charging voltages for the examined systems. Values of *n_eff_* have been determined at the same instant of time, *t* = 10 s, for charging and discharging conditions. The coefficient of energy efficiency increases, in general, with filler content and charging voltage. Energy-storing/retrieving efficiency reduces at the composites with higher filler content and at the maximum applied voltage level. The formation of some clusters, the lowering of potential barriers, and the enhanced conductivity of the systems can be considered as responsible. Optimum performance is observed for the 15 phr Fe_3_O_4_/10 phr BaTiO_3_/epoxy composite, reaching the value of 79.23%. Figure 9c presents the relative retrieved energy for all nanocomposites at the 100 V charging level, as a function of time. Relative energy is determined as the ratio of the retrieved energy from a specific hybrid composite upon the corresponding one of the epoxy resin, under the same conditions. Under the conditions shown in Figure 9c, optimum performance corresponds to the 50 phr Fe_3_O_4_/10 phr BaTiO_3_/epoxy composite. Its ability to retrieve energy is 12 to 16 times higher than that of the neat matrix.

**Figure 10:**
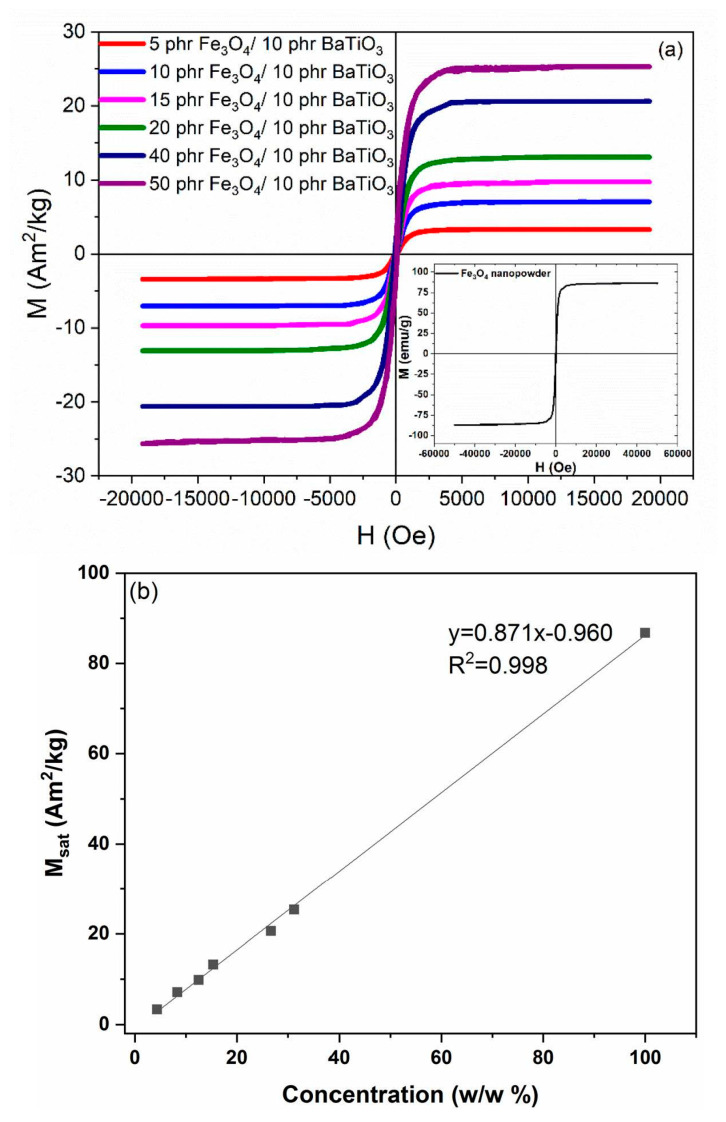
(**a**) Magnetic hysteresis loops for all hybrid composites, inset depicts the hysteresis loop of magnetite powder. (**b**) Magnetic saturation as a function of the Fe_3_O_4_ content.

Hysteresis loops of magnetization, for all studied nanocomposites, are depicted in Figure 10, at ambient temperature. Recorded spectra imply soft ferrimagnetic behavior for the nanocomposites, which is in accordance with the magnetic response of the ceramic nanoparticles of Fe_3_O_4_, inset of Figure 10a. Magnetic nanoparticles induce magnetic properties to the nanocomposites. Magnetization and magnetic saturation (*M_s_*) of the nanocomposites alter with magnetite content, tending to the values of neat Fe_3_O_4_ nanopowder. Interestingly, magnetic saturation (*M_s_*) follows a linear dependence on magnetic phase content, as shown in Figure 10b. The latter is considered an indirect indication for the fine dispersion of the iron oxide nanoinclusions in the epoxy resin [14].

In this study, hybrid epoxy composites with barium titanate microparticles and magnetite nanoparticles were fabricated and investigated, aiming to prepare multifunctional systems with improved and adjustable mechanical, thermal, electrical, and magnetic performance. Moreover, in these systems, electric energy can be stored and harvested providing an additional function to their performance. Multifunctional materials provide a suitable base for the development of systems exhibiting smart performance.

## 5. Conclusions

A set of hybrid composites x phr Fe_3_O_4_/10 phr BaTiO_3_/epoxy resin were fabricated and studied with varying magnetite contents. Structural and morphological characterization reveled the successful implementation of particles in the polymer matrix as well as their fine distribution. Magnetite is beneficial to both static and dynamic mechanical behavior, at least up to 20 phr content, thus defining the optimum concentration for the mechanical response. Glass transition temperature remains practically unaffected by filler content, implying balanced interactions between the particles and between particles and the matrix. Thermal stability, as resulted via TGA thermographs, increases with Fe_3_O_4_ content. Dielectric permittivity increases systematically with filler, apart from the system with the highest reinforcing phase content. The observed relaxation processes are: (i) IP, at the low-frequency and high-temperature edge, (ii) glass-to-rubber transition, at medium frequencies and temperatures, and (iii) re-orientation of polar side-groups, at high frequencies. Optimum dielectric behavior corresponds to the 40 phr Fe_3_O_4_/10 phr BaTiO_3_/epoxy resin hybrid composite. DRF spectra reflect the structural transition from the polar ferroelectric phase to the non-polar paraelectric phase of the barium titanate particles, which is clearly detectable in XRD patterns of the examined systems and particles. This transition can be exploited in systems with switchable polarization and dielectric response. Hybrid composites provide an appropriate base for the development of fast charge/discharge systems for storing and retrieving electrical energy. Coefficient of energy efficiency attains high values, 55.72% and 64.42%, for the system with the highest filler content, at the 50 and 100 V charging level, while optimum response corresponds to the 15 phr Fe_3_O_4_/10 phr BaTiO_3_/epoxy resin composite, reaching the value of 79.23% at the 150 V charging level. Finally, magnetic properties are induced in the hybrid composites by the magnetite nanoparticles. As expected, magnetic response alters with magnetic phase content. The system with the highest magnetic filler content obtains the value of 25.38 Am^2^/kg for magnetic saturation, while pure magnetic powder attains the value of 86.75 Am^2^/kg.

Gathering the results and conclusions derived from the studies on the various properties and behaviors, it can be understood that the occurring physical mechanisms synergistically construct the multifunctional performance of the systems. Properties and responses, individually induced by the constituents of the systems, tend to a global performance like the one described in Figure 1. Multifunctionality can be described or defined as the combination of different desirable properties in a material’s system, which should exhibit all necessary responses under various loading conditions at service. The developed and studied hybrid composites approach the performance of a material/device, being able to execute operations/functions under control.

## Figures and Tables

**Table 1:** Filler content in specimens, specimens’ mass and density, glass transition temperature determined via DSC and DMA, and the temperature corresponding to the 5% initial mass loss.

Filler Content in Specimens (phr)	Specimens’ Mass (g)	Specimens’ Density (g/cm^3^)	DSC	DMA	5% Mass Loss TGA
			*T_g_* (°C)	*T_g_* (°C)	*T* (°C)
Neat epoxy	1.8718	1.082	48.13	44.82	169.12
5 Fe_3_O_4_/10 BaTiO_3_	1.9141	1.174	41.65	46.71	187.60
10 Fe_3_O_4_/10 BaTiO_3_	1.8061	1.249	41.45	47.40	180.21
15 Fe_3_O_4_/10 BaTiO_3_	1.7390	1.284	40.33	46.75	191.10
20 Fe_3_O_4_/10 BaTiO_3_	2.3765	1.379	43.06	48.62	173.01
40 Fe_3_O_4_/10 BaTiO_3_	2.3266	1.411	41.02	43.70	176.51
50 Fe_3_O_4_/10 BaTiO_3_	2.1845	1.414	41.52	46.73	190.32

**Table 2:** Coefficient of energy efficiency, for studied systems, at three different charging voltages.

Filler Content in Specimens (phr)	*n_eff_* (%)		
	50 V	100 V	150 V
Neat epoxy	23.31	26.59	30.76
5 Fe_3_O_4_/10 BaTiO_3_	35.40	38.74	60.56
10 Fe_3_O_4_/10 BaTiO_3_	41.54	49.82	62.23
15 Fe_3_O_4_/10 BaTiO_3_	45.10	56.99	79.23
20 Fe_3_O_4_/10 BaTiO_3_	48.24	53.19	64.73
40 Fe_3_O_4_/10 BaTiO_3_	52.54	61.35	17.47
50 Fe_3_O_4_/10 BaTiO_3_	55.72	64.42	-

## Data Availability

Data are available upon request.

## Supplementary Materials

The following supporting information can be downloaded at: https://www.mdpi.com/article/10.3390/ma15051784/s1, Figure S1: (a) Energy-dispersive X-ray spectroscopy spectrum for the composite with 5 phr Fe_3_O_4_/10 phr BaTiO_3_ content. (b) SEM image from the composite with 50 phr Fe_3_O_4_/10 phr BaTiO_3_ content, at a lower magnification; Figure S2: (a) Real part of dielectric permittivity, (b) loss tanδ, and (c) σac as a function of frequency and temperature for the 10 phr Fe_3_O_4_/10 phr BaTiO_3_/epoxy hybrid composite.; Figure S3: Real part of permittivity as a function of temperature at 0.1 Hz, for all studied systems; Table S1: Filler content in specimens, specimens’ dimensions, mass, and density. Dimensions refer to the specimens for electrical measurements.
