# Supplementary material for: Multitasking Performance of Fe3O4/BaTiO3/Epoxy Resin Hybrid Nanocomposites
Source: Materials (Basel). 2022 Feb 26;15(5):1784. doi: 10.3390/ma15051784 (PMC8911469; doi:10.3390/ma15051784)
Supplement: Supplementary file 1 [file materials-15-01784-s001.zip › materials-1549979-Supplementary.pdf]

Multitasking Performance of Fe_3O_4/$BaTiO_3$/Epoxy Resin Hybrid Nanocomposites

Sevasti Gioti[1], Aikaterini Sanida[1], Georgios N. Mathioudakis[2], Anastasios C. Patsidis[1], Thanassis Speliotis[3], and Georgios C. Psarras[1,*]

[1]Smart Materials & Nanodielectrics Laboratory, Department of Materials Science, School of Natural Sciences, University of Patras, 26504 Patras, Greece,

[2]Institute of Chemical Engineering Sciences (ICE-HT), Foundation for Research & Technology-Hellas (FORTH), Stadiou Str., Platani, P.O.Box 1414, GR-26504 Patras, Greece,

[3]Institute of Nanoscience and Nanotechnology, NCSR "Demokritos", Aghia Paraskevi, 15310 Athens, Greece.

*Correspondence: G.C.Psarras@upatras.gr; Tel.: (+30 2610 996316)

Supplementary Information

Table S1. Filler content in specimens, specimens' dimensions, mass, and density. Dimensions refer to the specimens for electrical measurements.

Filler content in specimens (phr)	Specimens' mass (g)	Specimens' thickness (mm)	Specimens' diameter (mm)	Specimens' density (g/cm³)
Neat epoxy	1.8718	2.81	28.0	1.082
5 Fe_3O_4/10 $BaTiO_3$	1.9141	2.65	28.0	1.174
10 Fe_3O_4/10 $BaTiO_3$	1.8061	2.35	28.0	1.249
15 Fe_3O_4/10 $BaTiO_3$	1.7390	2.20	28.0	1.284
20 Fe_3O_4/10 $BaTiO_3$	2.3765	2.80	28.0	1.379
40 Fe_3O_4/10 $BaTiO_3$	2.3266	2.68	28.0	1.411
50 Fe_3O_4/10 $BaTiO_3$	2.1845	2.51	28.0	1.414

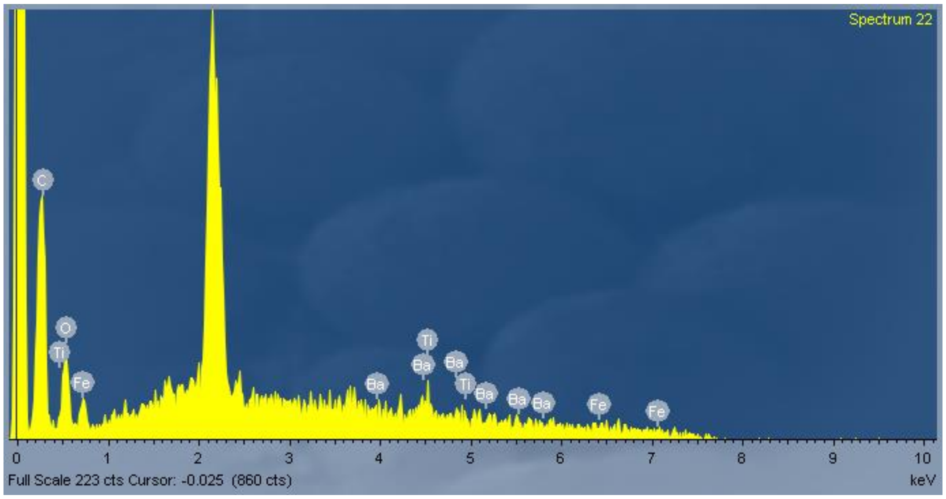

(a)

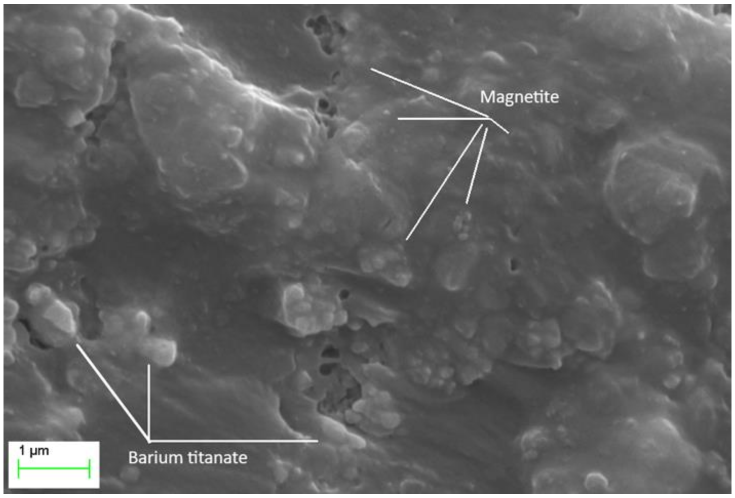

(b)

Figure S1. (a) Energy-dispersive X-ray spectroscopy spectrum for the composite with 5 phr Fe_3O_4/10 phr $BaTiO_3$ content. (b) SEM image from the composite with 50 phr Fe_3O_4/10 phr $BaTiO_3$ content, at a lower magnification.

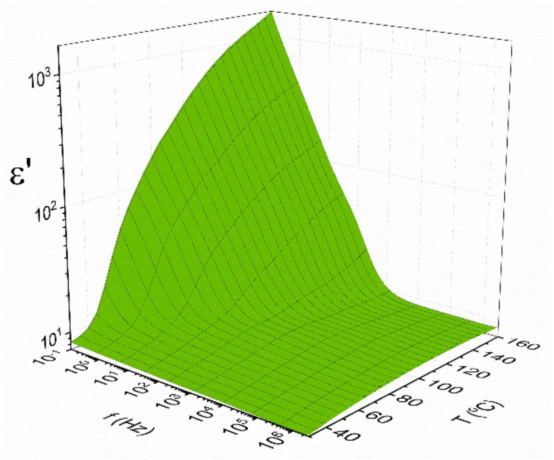

(a)

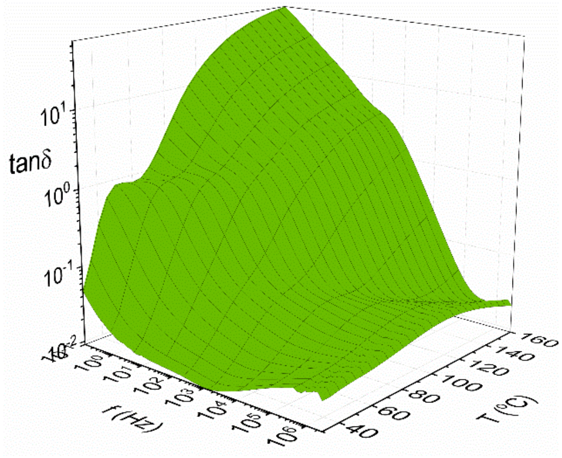

(b)

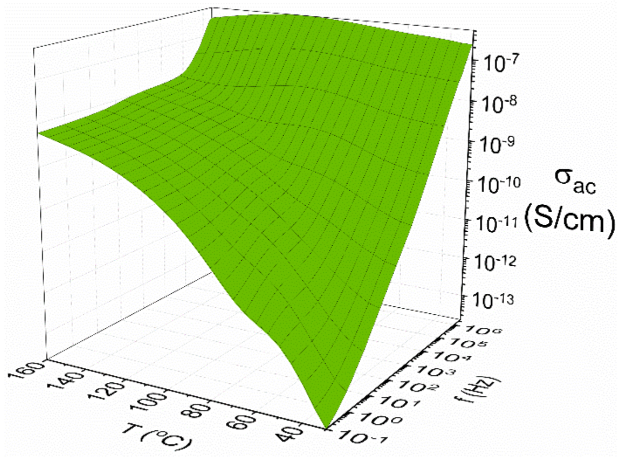

(c)

Figure S2. (a) Real part of dielectric permittivity, (b) loss tanδ, and (c) σ_{ac} as a function of frequency and temperature for the 10 phr Fe_3O_4/10 phr $BaTiO_3$/epoxy hybrid composite.

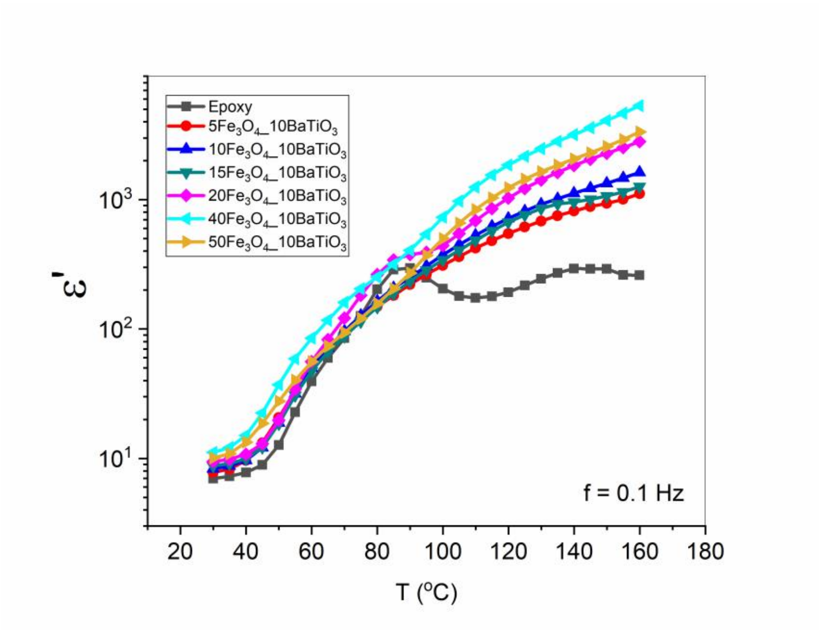

Figure S3. Real part of permittivity as a function of temperature at 0.1 Hz, for all studied systems.
